# Precision Surgery and Kidney Cancer: Knowledge of Genetic Alterations Influences Surgical Management

**DOI:** 10.3390/genes12020261

**Published:** 2021-02-11

**Authors:** Patrick T. Gomella, W. Marston Linehan, Mark W. Ball

**Affiliations:** Urologic Oncology Branch, National Cancer Institute, National Institutes of Health, Bethesda, MD 20892, USA; patrick.gomella@nih.gov (P.T.G.); wml@nih.gov (W.M.L.)

**Keywords:** renal cell carcinoma, kidney cancer, precision surgery, genetic alterations, Von Hippel-Lindau, hereditary leiomyomatosis and renal cell cancer, Birt-Hogg-Dubé

## Abstract

Renal cell carcinoma is a term that represents multiple different disease processes, each driven by different genetic alterations, with distinct histology, and biological potential which necessitates divergent management strategies. This review discusses the genetic alterations seen in several forms of hereditary kidney cancer and how that knowledge can dictate when and how to intervene with a focus on the surgical management of these tumors.

## 1. Introduction

In the United States, renal cell carcinoma (RCC) diagnoses are expected to reach over 73,000 new cases in 2020 with an estimated 14,800 deaths. RCC is the sixth most common cancer diagnosis in men and eighth most common in women [1]. Whereas RCC was once thought to be a single disease, it is now recognized to be multiple different entities. At least 17 genes have been identified that lead to the development of tumors arising in the kidney, each with different histologies and distinct biologic potential [2,3,4,5,6].

Hereditary kidney cancer is thought to account for 5–8% of all kidney cancer cases [7], though that number is likely an underestimate, with some studies estimating that up to 38% of RCCs have a hereditary component [8]. Numerous hereditary cancer phenotypes have been described that are associated with development of RCC, each with their own specific molecular alteration and typical clinical course. Many of the molecular underpinnings of sporadic RCC have been elucidated by studying hereditary kidney cancer. For instance, alterations of *VHL* first identified in families with von Hippel-Lindau are present in >90% of cases of sporadic clear cell RCC. Similarly, alterations in *MET* which were identified in patients with hereditary papillary renal carcinoma (HPRC) are also present in 17% of sporadic type 1 papillary RCCs, and 81% of these cases have altered *MET* status. Therefore, lessons from the management of hereditary kidney cancer have broad implications for the management of sporadic RCC.

In this review, we discuss the most prevalent hereditary kidney cancer syndromes, their clinical manifestations, genetic alterations, and treatment with a focus on surgical management. Knowledge of the genetic alterations of a kidney tumor, whether germline or somatic, allows for a precision surgical approach that can dictate both when and how to intervene.

## 2. Von Hippel-Lindau (VHL)

Phenotypic characteristics of von Hippel-Lindau syndrome were first described in the early 1900s relating specifically to angiomas of the eyes, cerebellum, and spine [9,10]. It was not until the late 1980s/early 1990s, via the evaluation of family clusters, that the *VHL* gene was localized to the short arm of chromosome 3 (3p25–26) [11]. The *VHL* gene is a tumor suppressor gene, with affected individuals inheriting a single mutated copy of the gene and a normal wild-type allele from the unaffected parent. Tumor formation is initiated when the wild-type allele undergoes an inactivating mutation or deletion in the affected target organs [12]. The *VHL* gene encodes for a protein that forms a complex with Elongin B, Elongin C, and Cullin-2. Under normoxia, this complex binds with hypoxia inducible factor (HIF) and targets it for ubiquitin-mediated proteasomal destruction. Without a functioning *VHL* protein and the associated degradation of HIF, the cell acts in a hypoxic state, leading to the upregulation of several angiogenic/cell cycle and proliferation pathways, such as increased VEGF, EPO, TGFaplha, and PDGFBeta [13,14]. Patients with a germline *VHL* mutation have over 90% disease penetrance by the age of 65. Manifestations include hemangioblastomas of the central nervous system, pheochromocytoma, cystadenomas of the epididymis, cysts and neuroendocrine tumors of the pancreas, endolymphatic sac tumors, and renal cysts/clear cell carcinoma [15]. Specific genotypes are associated with certain phenotypic manifestations, which can help guide screening and management of patients. Deletion and frameshift alterations have a higher propensity for development of clear cell RCC but low risk for pheochromocytoma. In contrast, missense alterations have a higher propensity for development of pheochromocytoma [16,17]. The median age of onset of RCC in patients with VHL is around 35 years of age [18], with approximately 25–60% of patients developing renal cysts and/or RCC in their lifetime [15]. The histologic subtype seen in patients with VHL is clear cell RCC.

## 3. Birt-Hogg-Dubé (BHD)

Originally described by three Canadian physicians in 1977 examining common skin findings in family members, Birt-Hogg-Dubé is an autosomal dominant condition, with the mutation mapped in 2001 to chromosome 17p11.2 [19] and the novel gene *FLCN* identified 1 year later [20]. *FLCN* is a tumor suppressor gene that encodes for the protein folliculin. Whereas its function has not been definitively elucidated, via its interaction with FNIP1, FNIP2 and AMPK, it has been shown to modulate mTOR signaling, TFE3 transcriptional activities, TGFB signaling, and PGC-1a mitochondrial biogenesis [21]. Clinical manifestations of this syndrome include fibrofolliculomas, lung cysts, and RCC. RCC onset is around 50 years of age, with approximately 30% of patients developing renal tumors [22]. Unlike VHL, BHD-associated RCC is associated with several different tumor histologies, with hybrid oncocytic (features of both chromophobe and oncocytoma) being the most prevalent, followed by chromophobe, oncocytoma, clear cell, and papillary subtypes [23].

## 4. Hereditary Papillary Renal Carcinoma (HPRC)

Hereditary Papillary Renal Carcinoma (HPRC) is an extremely rare autosomal dominant condition that can result in the development of multifocal and bilateral type 1 papillary renal tumors. Unlike the other syndromes described, HPRC is not associated with any clinical manifestations other than renal tumors, making its diagnosis difficult unless a high suspicion exists. Alteration of the *MET* gene (located on chromosome 7q31) was ultimately identified as the cause of this condition initially based on the study of families with high prevalence of papillary RCC without any identifiable changes on chromosome 3p [24,25]. Missense mutations are specific to the tyrosine kinase domain of the *MET* gene and act as a proto-oncogene. In normal cells, the interaction of MET kinase and its ligand, hepatocyte gross factor (HGF), results in phosphorylation of several tyrosines that are vital to cell proliferation and differentiation. In affected individuals, the activating mutation allows inappropriate upregulation of the numerous downstream regulators by the MET kinase without its interaction with HGF [26]. Penetrance is over 90% by age 60 and the median age of diagnosis of renal tumors is 57 years (range 46–63) [27].

## 5. Hereditary Leiomyomatosis Renal Cell Cancer (HLRCC)

Contrary to the indolent nature of HPRC, Hereditary Leiomyomatosis and Renal Cell Cancer (HLRCC) is associated with a more aggressive type 2 papillary RCC. HLRCC was first suggested as a hereditary syndrome in 1958, when dermatologists noted families with numerous cutaneous leiomyomas [28]. Its association with uterine leiomyomas (named Reed’s syndrome) was described in 1973 [29], and its association with the development of aggressive RCC was described in 2001. This coincided with the discovery of the associated germline mutation of the *FH* gene on chromosome 1q42.3–q43 [30,31]. *FH* encodes a Krebs cycle enzyme, fumarate hydratase, which is involved in the conversion of fumarate into malate. As tumor suppressor gene, HLRCC-affected cells are characterized by increased aerobic glycolysis and impaired oxidative phosphorylation. This alteration in glucose metabolism is an example of the Warburg Effect [32]. Additionally, a pseudohypoxic state develops with the inhibition of prolyl hydroxylase leading to increased HIF1a and its associated downstream effects [33]. The most common clinical manifestations in affected individuals include cutaneous leiomyomas in all genders and uterine leiomyomas in women, with high rates of early hysterectomy seen. Approximately 15% of those affected develop RCC over the course of their lifetime, and tumors have been seen in patients as young as 10 years old. These are aggressive tumors, and many patients present with locally advanced or distant disease even in the setting of small tumor sizes [34].

## 6. Succinate Dehydrogenase (SDH)-Deficient RCC

Just recently added to the World Health Organization classification of RCC [35], Succinate dehydrogenase (SDH)-deficient RCC is associated with a germline mutation in the genes encoding any of the *SDH* subunits (*SDHA*,* SDHB*,* SDHC*,* SDHD*). Initially described as a cause of hereditary paraganglioma/pheochromocytoma syndrome [36], SDH is another Krebs cycle enzyme that catalyzes the change of succinate to fumarate. Without a functioning *SDH* complex, succinate accumulates which leads to stabilization of HIF1a [37], creating a pseudohypoxic state and an increase in aerobic glycolysis similar to what is seen in HLRCC-associated tumors. Originally described in 2004, mutations in the SDHB subunit are the most frequently seen in SDH-deficient RCC [38]. ln addition to pheochromocytoma, paraganglioma, and RCC, patients are at risk for gastrointestinal stromal tumors. SDH-deficient RCC tends to present early (median age of 30 years in NCI series) and represent an aggressive disease with high potential for metastatic spread even at small sizes [39].

## 7. BAP1-Tumor Predisposition Syndrome (BAP1-TPS)

First implicated in the development of malignant mesothelioma and uveal melanoma, germline BRCA1-associated protein-1 (*BAP1*) mutations are a relatively recently discovered tumor predisposition syndrome [40]. The gene that encodes for BAP1 is located on chromosome 3p21.1 and functions as a de-ubiquinating enzyme, with functions related to cellular integrity via cell cycle control and DNA damage repair. Recently, its role in apoptosis was described via stabilizing the type 3 inositol-1,4-5, triphosphate receptor, which modulates calcium release into the cytosol. Decreased levels of BAP1 lead to decreased fidelity of DNA damage repair in the nucleus along with decreased triggering of cell death when DNA damage has accumulated [41,42]. In addition to mesothelioma and uveal melanoma, patients are predisposed to develop cutaneous melanomas and RCC [43]. Examination of families with a *BAP1* mutation and RCC showed the development of early onset tumors, many of which were fast growing and with higher Fuhrman grade on pathologic analysis [44]. Whereas less than 100 families with this syndrome have been described, the frequency of RCC is reported to be 3–10% [45,46]. Somatic *BAP1* loss in sporadic RCC has been linked to poor prognoses [47,48]. This fact, along with early onset and higher grade tumors in patients with germline mutation, suggest that the renal cancers seen in this syndrome are potentially more aggressive.

## 8. Tuberous Sclerosis Complex (TSC)

TSC is a multisystem disorder first described in the late 19th century [49]. It is inherited in an autosomal dominant fashion with mutations mapped in the 1990s to the *TSC1* and *TSC2* genes located on chromosome 9q34 and 16p13, respectively [50,51]. *TSC1* and *TSC2* are tumor suppressor genes that follows Knudson’s two-hit model with development of the manifestations occurring with loss of the 2nd hit mutation/inactivation. *TSC1* encodes the protein hamartin and *TSC2* encodes the protein tuberin, which together form heterodimers to interact with several proteins [52]. Its primary downstream effect appears to inhibit the mammalian target of rapamycin (mTOR) pathway, which plays a role in cell proliferation and growth. Clinical manifestations primarily develop in the skin, central nervous system, eyes, lungs, and kidneys, with some patients developing seizures or mental retardation. The most noticeable changes are skin changes, including the development of hypopigmented macules, shagreen patches, facial angiofibroma, and ungual fibroma. Renal manifestations are primarily the development of bilateral multifocal cysts and angiomyolipoma [53]. Whereas the risk of RCC appears to only be slightly higher than the general population with a rate of about 3–5%, patients with TSC appear to develop malignant tumors at an earlier age. Series of TSC-related RCCs have shown that mean ages are between 30–42 years, and there is a slight female predominance [54,55]. Kidney cancers seen represent a wide range of histologies, with clear cell, papillary, chromophobe, hybrid tumors, and oncocytomas all being reported [53,55]. 

## 9. Clinical Management

The first step in precision management of these cancers remains a high clinical suspicion for a hereditary cancer syndrome to trigger appropriate genetic counseling and potential germline testing prior to planned surgery. Besides the more obvious clinical symptoms of the syndromes with visually apparent clinical manifestations, age at diagnosis and family history are important clues to prompt a genetic evaluation. Age at diagnosis of less than 46 is proposed as an age cutoff to prompt germline testing [18]. Additionally, the presence of bilateral/multifocal disease and/or a family history of RCC should prompt the need for genetic evaluation. At minimum, a detailed history focused on manifestations of hereditary syndromes, a family pedigree, and a physical examination focused on cutaneous manifestations of these syndromes should strongly be considered. Whereas some urologists may feel comfortable evaluating for these syndromes, there should be a low threshold for referral to a genetic counselor. This early suspicion can allow for genetic evaluation and diagnosis prior to any planned surgery so that germline mutations discovered can guide the surgical approach and planning.

The mainstay of treatment of hereditary RCC is nephron sparing surgery for localized disease and systemic therapy for metastatic disease. The goals of treatment are to minimize the risk of metastasis, to minimize the number of repeat ipsilateral renal surgeries over a patient’s life, and to maximize renal preservation when possible. For localized disease, decades of experience managing these patients have made it possible to tailor the approach based on the specific germline mutation as a direct result of the biological nature of the renal tumors. Biologic aggressiveness is associated with each specific germline mutation. Similarly, growth rates are providing additional data to help guide management. In a study of 292 genetically defined patients, growth rates were analyzed from 435 tumors. Bap1-deficient tumors had the fastest growth rate (median 0.6 cm/year), while tumors with MET-activated tumors had the slowest growth rate (0.015 cm/year) [56].

## 10. Precision Surgery: When to Intervene

Several hereditary syndromes are managed with an initial period of active surveillance until the largest solid tumor reaches 3 cm. Longitudinal follow-up has shown this to be a safe strategy in patients with VHL, HPRC, and BHD, and this strategy has been extrapolated to TSC [57]. Growth rates help guide the frequency of active surveillance of renal lesions, especially in VHL patients who undergo imaging every 12–36 months based on their established tumor growth and current size. In general, patients with no current renal tumors are surveilled less frequently, up to every 36 months, while those with larger tumors are reimaged more frequently. BAP1-altered tumors often exhibit faster growth and have been associated with high grade, high stage, and low survival. Whereas active surveillance can be utilized, it is likely prudent to image these tumors more frequently. This active surveillance approach is not recommended in patients with HLRCC or SHD-deficient tumors, and immediate surgery is the preferred strategy. Surgery is the primary recommended management of localized kidney cancer, with nephron sparing surgery preferred for small renal masses and patients with hereditary syndromes given the lifelong risk of tumor development and potential need for numerous surgical resections [58]. Table 1 illustrates the differences in our surveillance approaches for several hereditary kidney cancer syndromes.

## 11. Precision Surgery: How to Intervene

**Figure 1:**
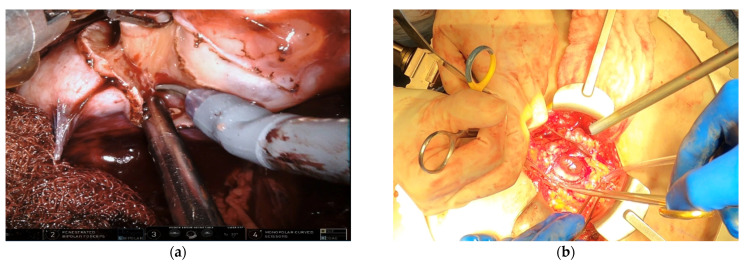
(**a**) Intraoperative view of a robotic tumor enucleation performed on a patient with von Hippel-Lindau (VHL) syndrome. Note: The plane of dissection is between the normal renal parenchyma and tumor pseudocapsule. (**b**) Intraoperative view of an open partial nephrectomy with wide excision performed on a patient with Hereditary Leiomyomatosis Renal Cell Cancer (HLRCC). Note that the plane of dissection is approximately 1 cm wider than the base of the tumor.

Surgical management of hereditary syndromes can vary in several respects. The most notable are the type of surgical approach (open or minimally invasive), and the extent of resection (tumor enucleation or wide excision). Figure 1 shows an example of the difference between a minimally invasive tumor enucleation for VHL and an open wide excision partial nephrectomy for HLRCC.

For less biologically aggressive tumors, such as those seen VHL, HPRC, TSC, and BHD, our default approach is minimally invasive partial nephrectomy with enucleation of the renal tumors. This approach maximizes normal renal tissue preservation. Enucleation is performed by incising the renal capsule circumferentially around the tumor and gently separating the normal parenchyma from the tumor capsule. Whereas most of the dissection is performed bluntly, if perforating blood vessels are encountered, they can be controlled with cautery and cut or cut sharply and suture ligated later. If the true enucleation plane is entered and followed, bleeding tends to be minimal, especially in the setting of pneumoperitoneum, and many of these enucleations can be performed without hilar clamping. One important point regarding enucleation is that sometimes patients lack a robust pseudocapsule. In general, VHL tumors have a robust pseudocapsule, while those in HPRC and BHD are sometimes more fragile. In this setting, a small normal margin can be taken if appropriate to help prevent tumor spillage [59].

HLRCC, SDH, and BAP1 are associated with more aggressive tumors. HLRCC specifically can have infiltrating tumor cells into normal parenchyma that cannot be appreciated with imaging or on intraoperative examination. Partial nephrectomy, if performed, requires a margin of normal parenchyma, and intraoperative frozen sections should be considered to ensure adequate resection has been performed. Open approaches should be strongly considered given the increased tactile feedback to help minimize risk of tumor spillage [59]. If a wide margin cannot be obtained because of the size or depth of a tumor, one should consider radical nephrectomy, particularly for HLRCC.

Removal of locoregional lymph nodes at time of surgery for RCC continues to be controversial. Results of the only randomized trial to date (EORTC 30881) did not show a difference in either progression free survival or overall survival between patients undergoing lymph node dissection at time of radical nephrectomy [60]. Critics of this trial discuss the higher rates of low-risk patients included and overall low lymph node yields. The histopathologic diagnoses for the patients in the trial were not included, but a reasonable assumption is that the majority were sporadic clear cell RCC patients. The role of lymph node dissection in a patient with a hereditary germline mutation is even less clear. Given the aggressiveness of the tumors seen in certain germline mutations (specifically HLRCC and SDH-deficient tumors), we routinely perform a local regional lymph node dissection at time of surgery in these settings for larger or more complex solid tumors, even in the setting of preoperatively normal lymph nodes.

## 12. Conclusions

Knowledge of a patient’s germline genetic alterations have multiple implications. Not only can it dictate screening for the patient and their family, but it is imperative in delivering a precision surgical approach that can maximize oncologic benefit while minimizing surgical morbidity. Knowledge of when to employ active surveillance and when to resect, when a minimally invasive approach is preferred and when an open operation is required, and the extent of a margin to take is directly inferred from a patient’s germline or tumor’s somatic alteration.

## Figures and Tables

**Table 1:** Examples of lesions seen in several hereditary kidney cancer syndromes, with difference in initial active surveillance and surgical approach noted.

Germline Alteration	BHD	VHL	BAP1	HLRCC
	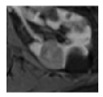	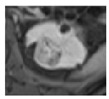	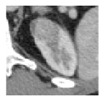	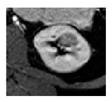
Tumor size	2.5 cm	2.5 cm	2.5 cm	2 cm
Active Surveillance versus Surgery	Active surveillance	Active surveillance	Surgery or Active Surveillance	Immediate Surgery
Next Imaging Follow-up	24 months *	12 months *	6 months if surgery deferred	Immediate Surgery
Surgical Approach	Robotic surgery	Robotic surgery	Robotic surgery	Strongly consider open surgery
Extent of Resection	Tumor enucleation	Tumor enucleation	Partial nephrectomy with margin	Partial nephrectomy with wide margin or radical nephrectomy. Consider regional lymph node dissection, especially for larger and centrally located tumors

* Depending on established growth rate, imaging follow-up can be shortened or extended.
